# Generating Dual-Mode Dual-Polarization OAM Based on Transmissive Metasurface

**DOI:** 10.1038/s41598-018-36677-6

**Published:** 2019-01-14

**Authors:** Xin Qi, Zheyuan Zhang, Xianzheng Zong, Xiaofeng Que, Zaiping Nie, Jun Hu

**Affiliations:** School of Electronic Science and Engineering, University of Electronic Science and Technology of China, Chengdu, 610054 China

## Abstract

Recent advances in electromagnetic (EM) waves with helical phase wave-front carrying orbital angular momentum (OAM) has drawn great attention, since it is believed to be a promising candidate for the next generation of wireless communication technology. To make the design more practical, here, a transmissive metasurface for generating dual-mode and dual-polarization OAM has been designed, manufactured and experimentally validated. To generate EM waves carrying OAM, the element structure is well-designed and can introduce additional phase to the incident wave. The employed four-layer cascaded metasurface demonstrates a high performance of transmission and complete phase control. Dual-mode operating characterization is realized by applying the polarization-dependent physical response. Moreover, experimental results including near-field and far-field properties are conducted to validate the numerical simulations. The proposed method in this paper promotes the practical design and realization of OAM vortex waves for the next generation of wireless communication technology.

## Introduction

With the modern wireless communication technologies developed widely and rapidly, its data capacity is reaching theoretical limit. Electromagnetic (EM) waves carrying orbital angular momentum (OAM) have been explored to enhance the system capacity due to its extra degree of freedom of angular momentum^1,2^. The spiral phase front is believed to carry additional information to provide extra data capacitance, and variety of studies on OAM have been reported in both domains of radio and optics^3–5^. Recently, applications based on OAM have been introduced in wireless communication^6,7^, optical manipulation^8,9^, target detection^10^ and microwave imaging^11,12^. Approaches for generating OAM have been developed well in recent years, such as spiral phase plate^13–15^, spiral reflectors^5^, circular waveguide^16^, and uniform circular antenna arrays^17–20^. Among them, the first method is originated from the optics, which has simple structures and is easy to be implemented. On the contrary, the spiral reflector is difficult to be manufactured. Antenna arrays need a complex feeding system and the number of elements limits the mode number. A new form of OAM-carrying EM wave that propagates along the transverse direction is generated by using traveling-wave circular slot antenna^21^. All methods mentioned above are hard to generate dual-mode OAM simultaneously. Recently, some traditional antennas like cylinder dielectric resonator^22^, circular slot antenna^23^, and patch antenna^24–26^ have been proposed to generate multiple OAM modes at the same time, however, a complicated exciting condition or feeding system prohibits their practical applications. Metasurface, as a powerful solution to manipulate the wave front of reflected or transmitted EM waves, has been studied and designed to control EM phase to generate vortex waves carrying OAM, characteristics of easy fabrication and free of complicated feeding systems are promising for their practical engineering applications, however, most of the reported work are based on the reflective metasurfaces and not compact enough due to the existing of air layer^27–32^.

**Figure 1:**
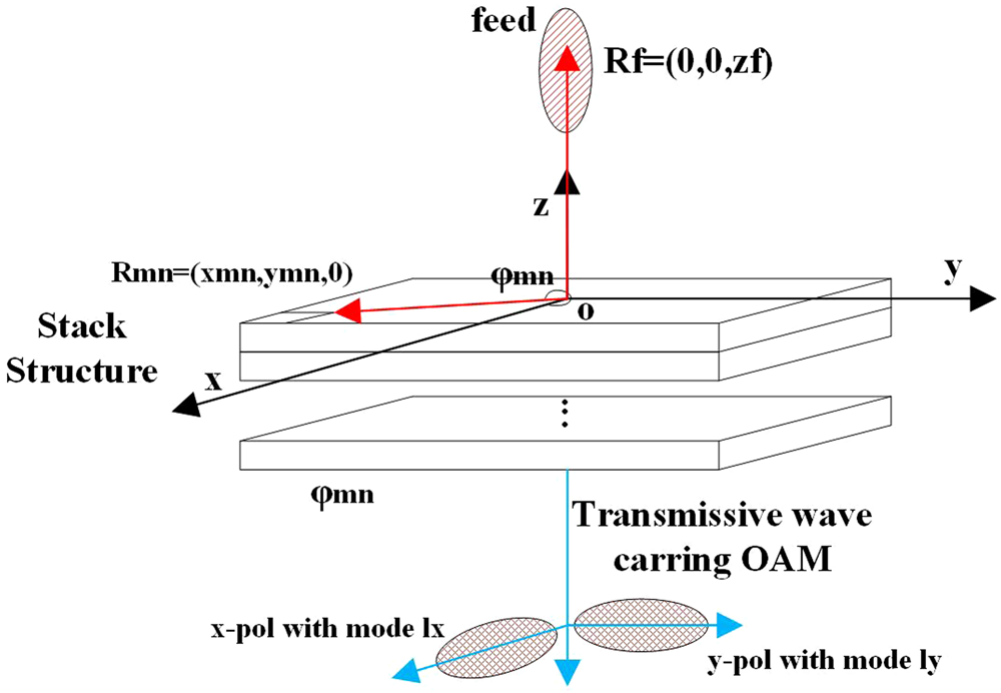
Working mechanism of OAM-generating transmissive metasurface.

In this paper, a transmissive metasurface is designed, manufactured and experimentally validated to generate dual-mode dual-polarization OAM vortex waves simultaneously. The proposed OAM generator consists of a cascaded metasurface which has four metallic layers and three dielectric layers, and an illuminating feed system. The whole process is schematically illustrated in Fig. 1. The high transmission efficiency and complete phase control are maintained by a well-designed element cell and the cascaded structure. To achieve a dual-mode operating system at the same time, a general strategy to control the operating OAM mode is proposed by tuning two orthogonal polarizations independently. For experimental demonstration, a C-band (frequency is 7.5 GHz) transmissive metasurface operating with dual-polarization and dual-mode of 2 and 4 respectively is fabricated and tested. The proposed method promotes the practical engineering applications of OAM, especially in wireless communications technology.

## Results

### Element design and working mechanism of dual-mode dual-polarization

When a monochromatic wave impinges on a metasurface consisting of global mirror symmetry elements with respect to $x\to -\,x$ and $y\to -\,y$, the EM response can be described by the Jones’ matrices R and T as Equations (1) and (2), where (x,y) is the location of the element center,*r*_*xx*_, *r*_*yy*_, *t*_*xx*_, *t*_*yy*_, respect the reflection and transmission coefficients^33^.

1 $$R(x,y)=(\begin{array}{c}{r}_{xx}\,\,\,\,0\\ 0\,\,\,\,\,{r}_{yy}\end{array}),$$

2 $$T(x,y)=(\begin{array}{c}{t}_{xx}\,\,\,\,0\\ 0\,\,\,\,\,{t}_{yy}\end{array}),$$

In our analysis, the reflection coefficient $R=0$ while the transmission coefficient $T=1$. The transmissive phases ${\phi }_{xx}$ and ${\phi }_{yy}$ are controlled by tuning the element structures at x- and y-directions respectively, and at the same time, the amplitudes of transmissive coefficients should be kept as 1 as much as possible. Due to the orthogonal property of x- and y-polarizations, a high isolation can be achieved between different OAM modes, which is more advantageous than the reported work^30^.

**Figure 2:**
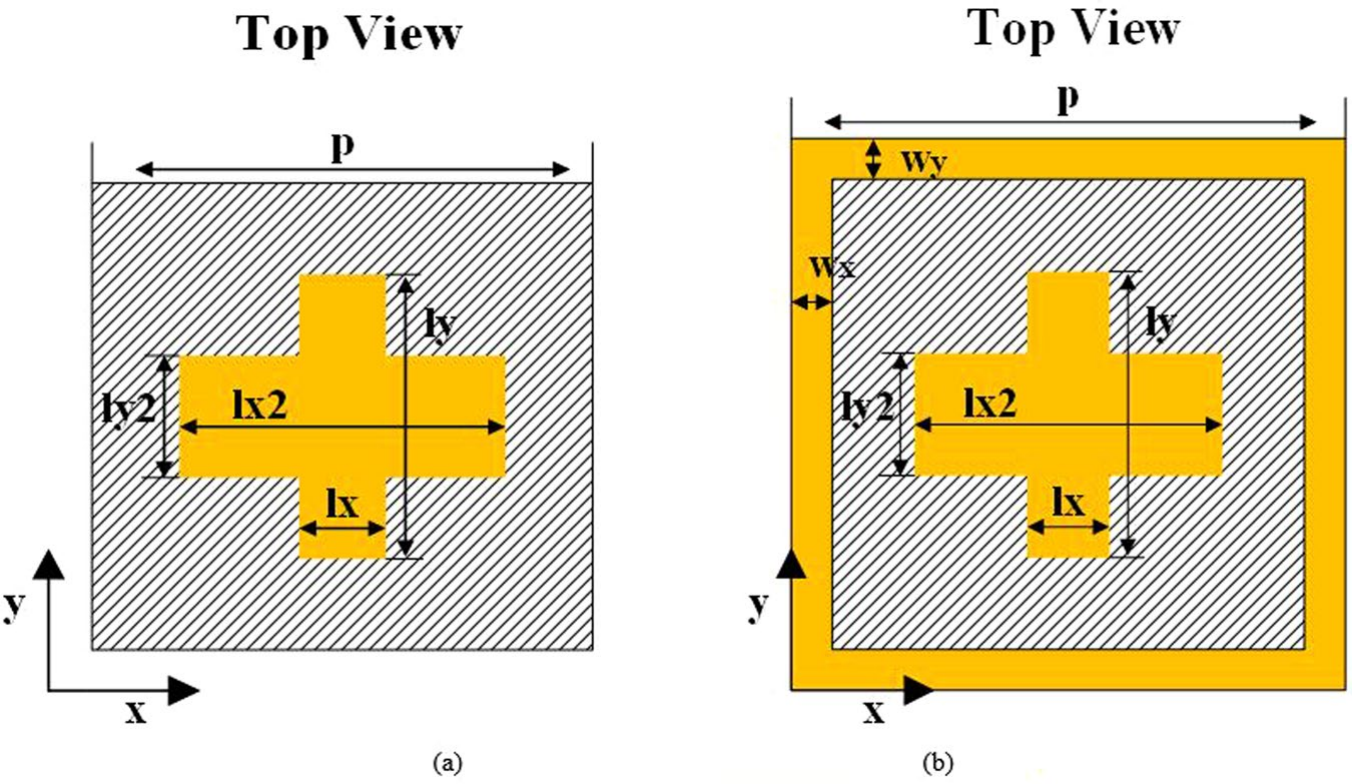
Cross-type element (**a**) without square loop (**b**) with square loop.

**Figure 3:**
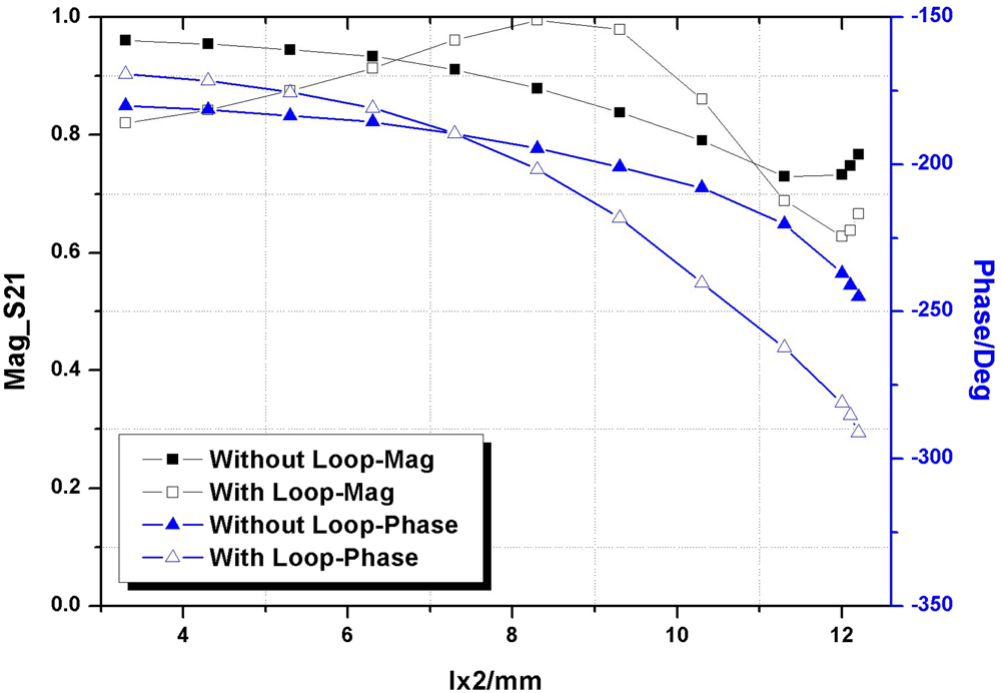
Magnitude of S21 and phase versus length of lx2 for element structures with and without square loop.

To keep the element totally transparent, a cascaded structure with identical elements in all layers is used commonly^33–35^. Tradeoffs between transmissive amplitude, phase control and fabrication complexity are made by adjusting the number of layers. Here, we choose a four layer design to maintain a perfect transmission and complete phase coverage. A general strategy to control two orthogonal polarizations independently is to apply cross-type patches, as shown in Fig. 2(a). Unfortunately, a single cross-type design is hard to balance the performance of transmission amplitude and phase coverage. Therefore, a square loop has been introduced in our element design, which provides a new freedom to cover a 360° phase-variation range and enhance transmission, as illustrated in Fig. 2(b). Simulation results for one layer structure with and without square loop are compared in Fig. 3. It can be found that the phase variation range has been increased to 122° from 44° by introducing the square loop. The incident wave is x-polarization with the frequency of 7.5 GHz and the length of ly is set as 9 mm. Other common parameters after optimizing for both structures are listed in Table 1. The substrate is F4B (${\varepsilon }_{r}=2.65$, $\tan \,\delta =0.001$). All full-wave EM simulations in this section are conducted by using CST Microwave Studio^36^. The excitation is a plane wave that normally incidents upon the periodic metasurface.

**Table 1:** Common parameters for element structure (Units: mm).

p	wx = wy	lx = ly2	td
14	0.6	3.3	1.5

**Figure 4:**
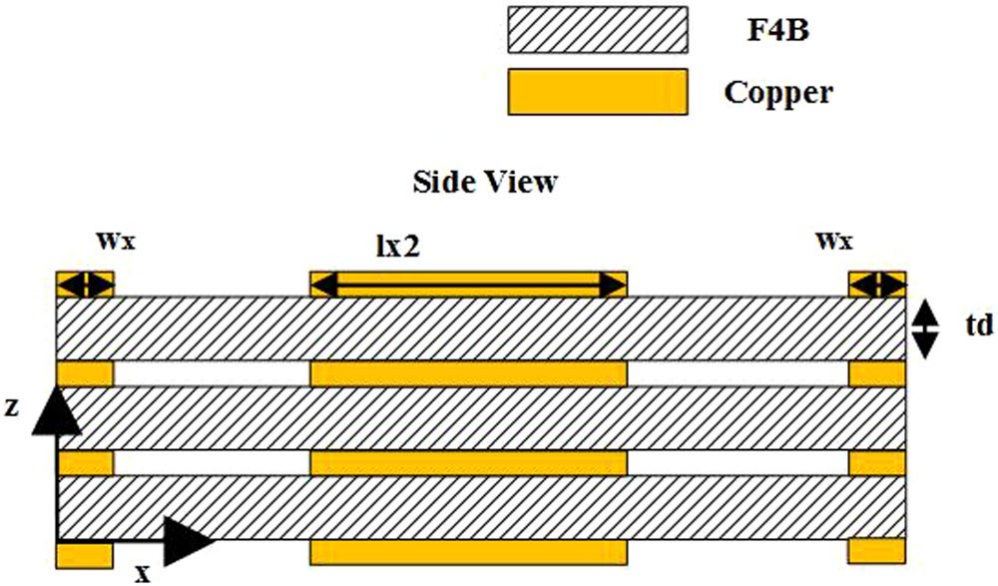
Side view of the proposed element structure.

Adjusting the number of layers, the optimized range of phase variations for two structures shown in Fig. 2 are summarized in Table 2. Obviously, the proposed element structure with square ring has a better performance than the original design, and with the number of layers increasing, the range of phase variations increases and a 360° transmission phase coverage is fulfilled. A side view of the final element structure is shown in Fig. 4.

**Table 2:** Range of phase variations for different layers (Units: degree).

Layer Number	With loop		Without loop	
	Range	Abs Value	Range	Abs Value
One layer	(−291, −169)	122	(−224, −180)	44
Two layers	(−422, −181)	241	(−387, −204)	183
Three layers	(−663, −193)	470	(−554, −226)	328

**Figure 5:**
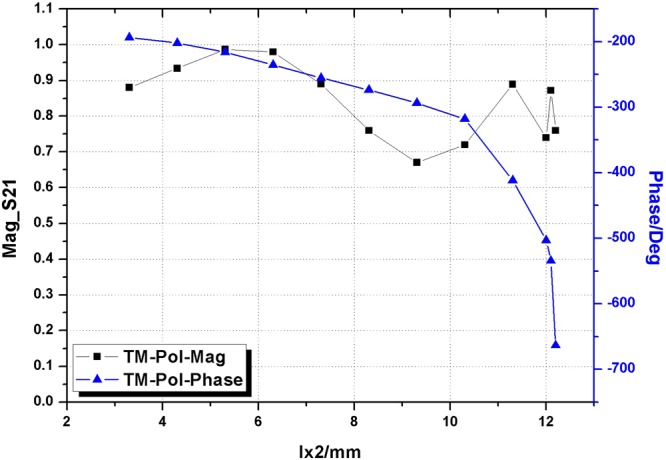
Magnitude of S21 and phase variation versus length of lx2 for TM.

**Figure 6:**
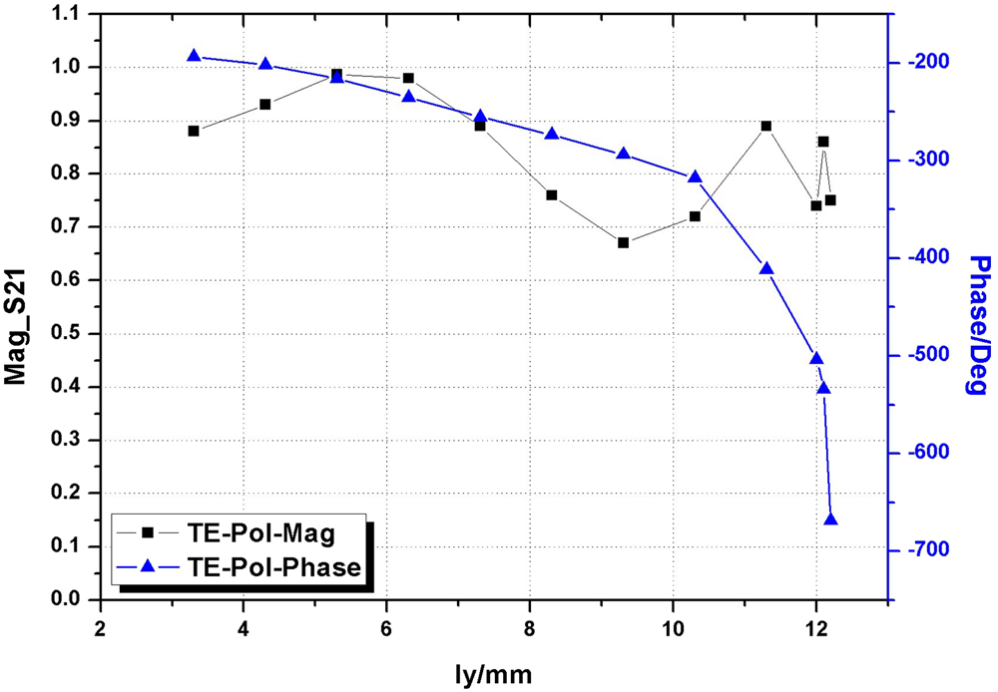
Magnitude of S21 and phase variation versus length of ly for TE.

Furthermore, two different polarizations are investigated for the structure shown in Fig. 4. For x-polarization (TM-Pol) ly is set as 9 mm and for y-polarization (TE-Pol) lx2 is set as 5 mm. Results of transmission coefficients and phase variations for TM- and TE-polarizations are pictured in Figs 5 and 6 respectively.

Due to the orthogonal property of element structure, two different polarizations corresponding to two OAM modes can be tuned independently and a high isolation can be realized. It can be found that the proposed metasurface is able to provide a high performance of transmission and complete phase control for both polarizations, i.e., the average magnitude keeps a transmittance greater than 80% while the phase covers an 360° variations.

### Transmissive metasurface design and numerical results

To generate an OAM beam, an azimuthal phase of ${e}^{jl\phi }$ needs to be introduced to the transmissive wave, where $l$ is the mode number and $\phi$ is the azimuthal angle. For the mn-th element of the whole transmissive metasurface, the required compensating phase can be obtained by the following equation^30^,

3 $${\varphi }_{mn}=-{2\pi /\lambda }_{0}|{{\bf{R}}}_{mn}-{{\bf{R}}}_{f}|+l{\phi }_{mn},$$

where **R**_*mn*_ and **R**_*f*_ are position vectors of the mn-th element and effective phase center of the feeding antenna, ${\lambda }_{0}$ is the wavelength of freedom.

**Figure 7:**
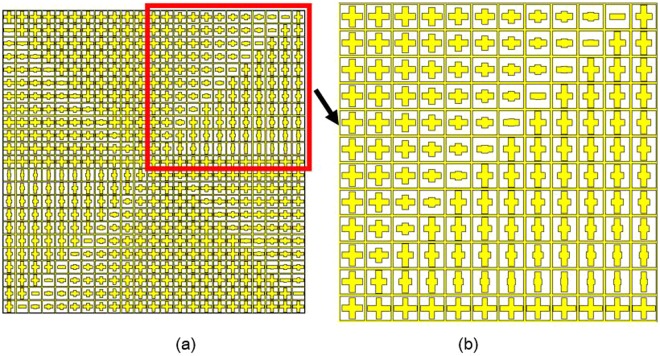
Configuration of the designed transmissive metasurface.

**Figure 8:**
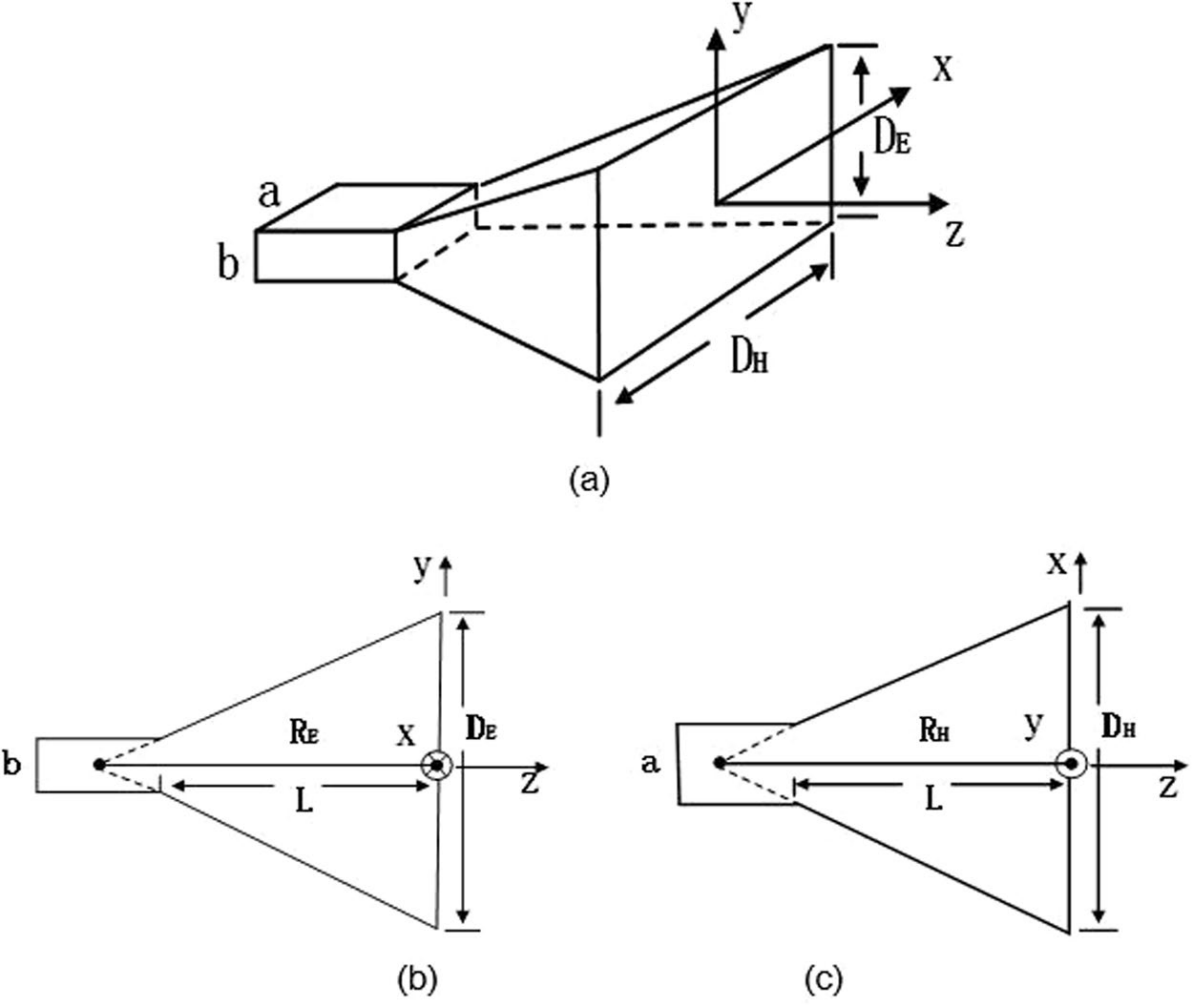
(**a**) Geometry of pyramid horn antenna (**b**) view of E-plane (**c**) view of H-plane.

After optimizing the element structure, the final transmissive metasurface with 23*23 elements is built to generate dual-mode dual-polarization OAM beam at the frequency of 7.5 GHz. Dual modes are 2 and 4 corresponding to x-pol and y-pol respectively. The final configuration of the design is a square array with the dimension of 322 mm*322 mm, as illustrated in Fig. 7(a), detail structure in red frame is shown in Fig. 7(b). To generate two orthogonal polarized incident waves at the same time, a pyramid horn antenna is designed specially and applied as the feeding source. It incidents the metasurface in normal with a 45° offset from the x-axis, so that the x- and y- polarizations of the system are excited equally. The geometry model of a pyramid horn antenna is shown as Fig. 8(a), views of E-plane and H-plane patterns are shown in Fig. 8(b,c), and parameters of the designed horn antenna are summarized in Table 3.

**Table 3:** Parameters of the designed horn antenna (Units: mm).

a	b	R_E_	R_H_	D_E_	D_H_
28.499	12.624	243	288	48.5	75.9

**Figure 9:**
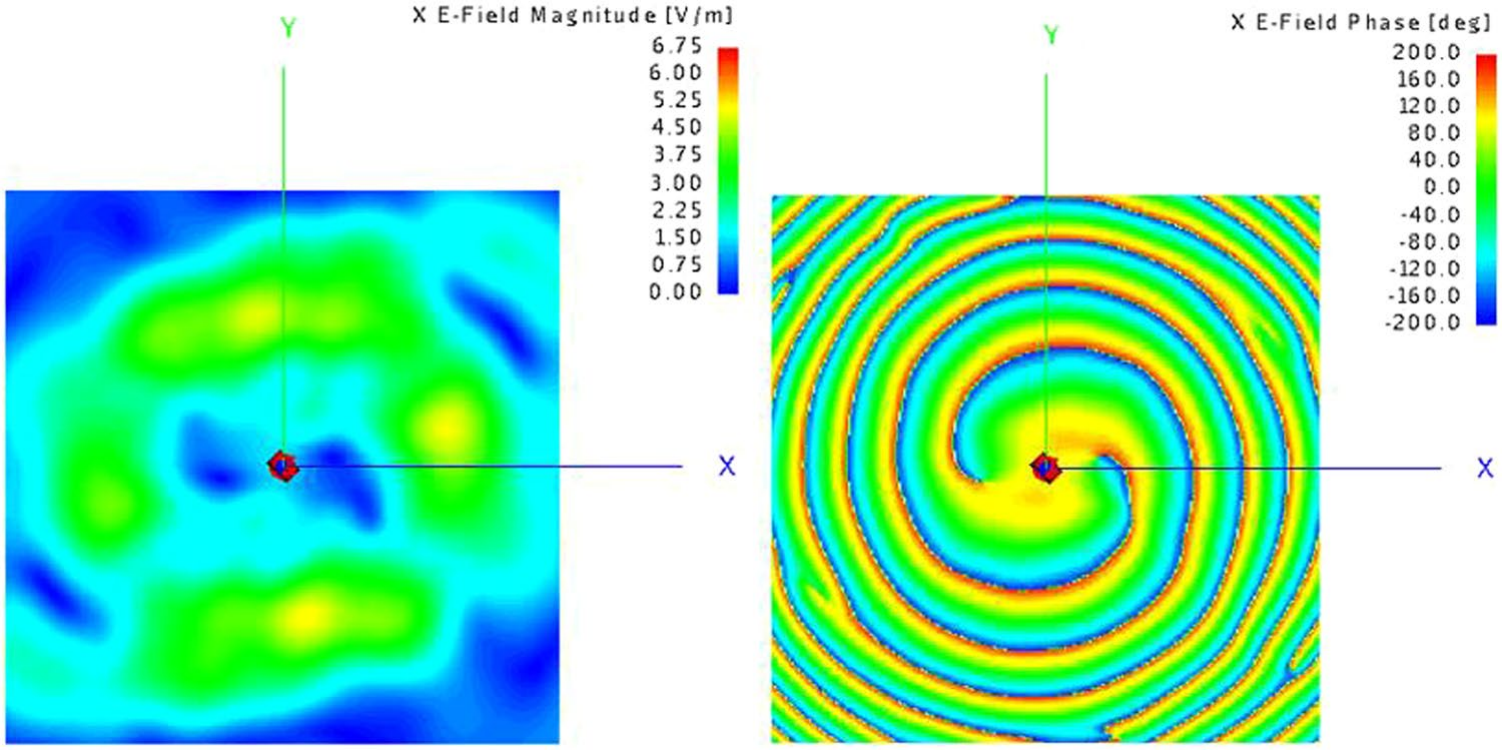
(**a**) Magnitude of electrical field for x-pol with mode = 2 (**b**) Phase of electrical field for x-pol with mode = 2.

**Figure 10:**
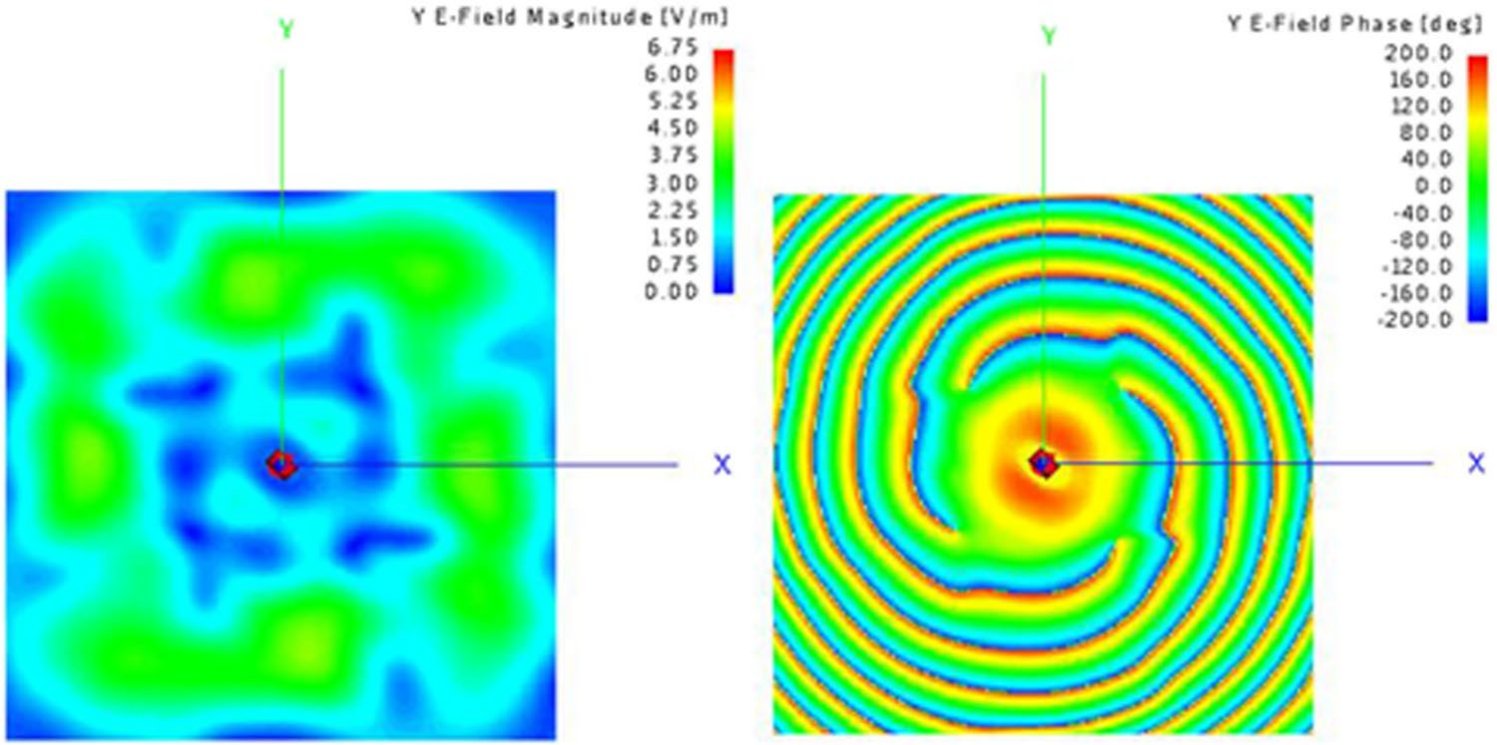
(**a**) Magnitude of electrical field for y-pol with mode = 4 (**b**) Phase of electrical field for y-pol with mode = 4.

To maintain the main energy impinges on the metasurface and eliminate the truncation influence resulted from the edges of the metasurface, the horn aperture is 0.3 m away from the metasurface and the near-field sampling plane is 0.6 m away from the metasurface. Numerical results simulated by Altair FEKO which is based on the Method of Moments^37^ are shown in Figs 9 and 10.

Figures 9(a) and 10(a) reveal a magnitude null at the center of beam clearly, and Figs 9(b) and 10(b) display perfect phase variations corresponding to OAM mode number of 2 and 4 respectively. The presented simulation results demonstrate that dual-polarization dual-mode OAM vortex wave can be generated simultaneously and effectively by the proposed transmissive metasurface.

### Discussions about frequency shifting influence, polarization isolation and OAM modes purity

**Figure 11:**
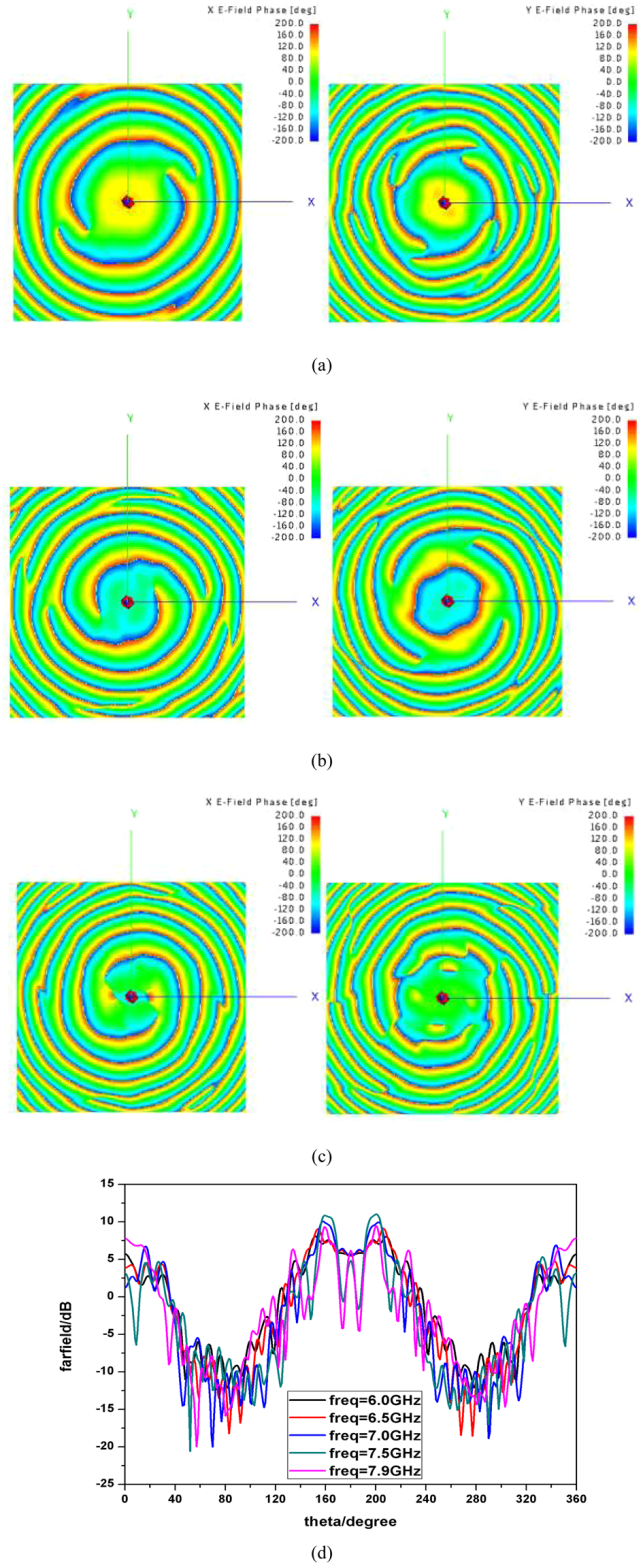
(**a**) Frequency is 6.0 GHz, mode = 2(left), mode = 4(right). (**b**) Frequency is 7.0 GHz, mode = 2(left), mode = 4(right). (**c**) Frequency is 7.9 GHz, mode = 2(left), mode = 4(right). (**d**) Far-field radiation patterns of y-o-z plane for different frequencies.

The influence of frequency shifting on the generation of OAM beams are investigated in the following. Phase distributions of near-field and far-field patterns for different frequencies are shown in Fig. 11. It can be found that the near-field characteristics keep stable when frequency changes, while for far-field the total gain decreases when frequency-shifting happens. The designed properties work well from 6.0 GHz to 7.9 GHz. Work for broadband or multiband OAM will be discussed in the future.

**Figure 12:**
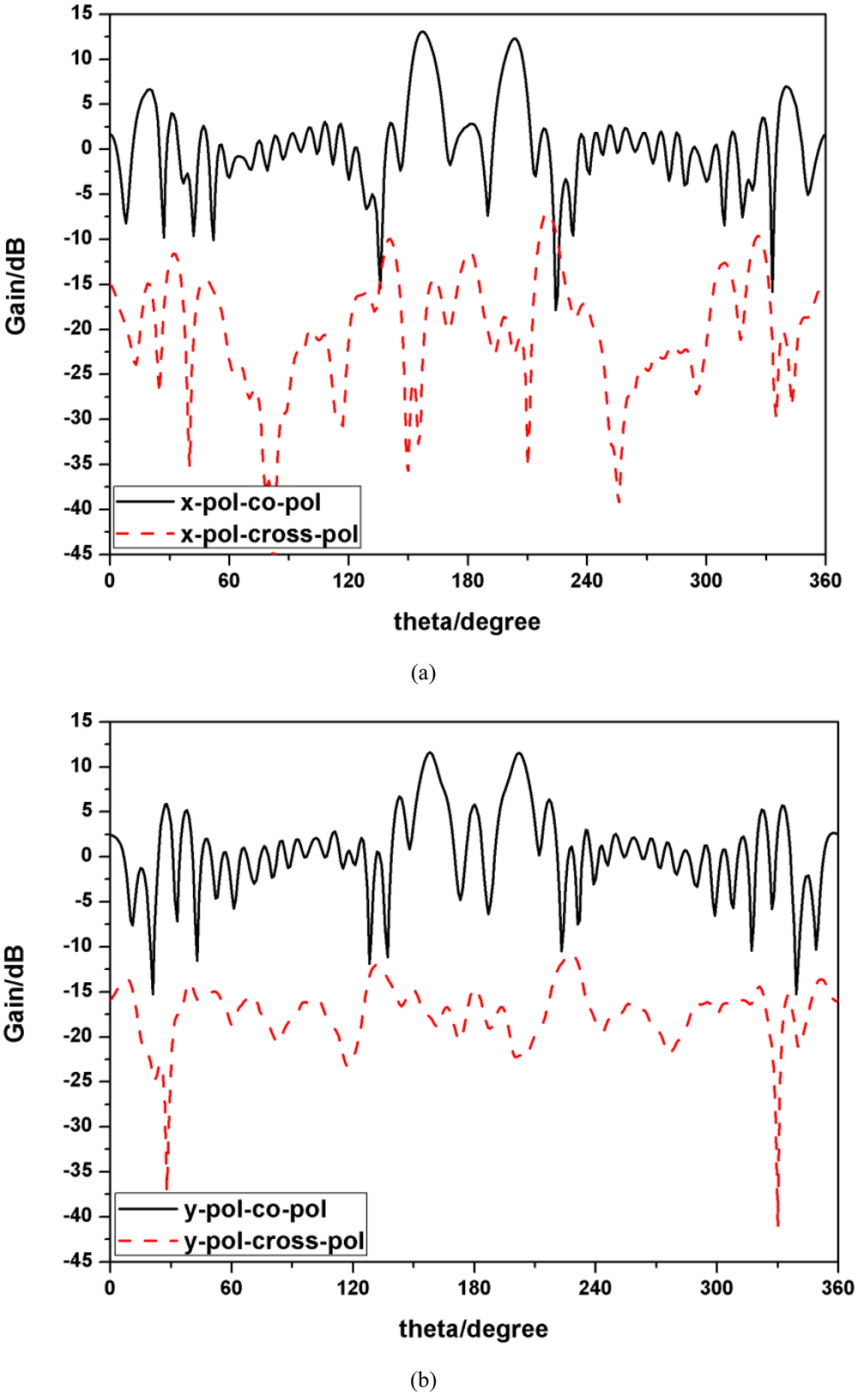
(**a**) Far-field radiation patterns of co- and cross-polarizations for x-polarized incident wave. (**b**) Far-field radiation patterns of co- and cross-polarizations for y-polarized incident wave.

In the following, properties of polarization isolation and OAM modes purity are analyzed in detail. The simulated far-field radiation patterns of co- and cross-polarizations are shown in Fig. 12(a,b) corresponding to x-polarized incident wave and y-polarized incident wave respectively. It is observed that the isolations for different polarizations are larger than 20 dB and a good polarization isolation has been achieved. Since the mechanism of the dual-mode operation is realized by applying the polarization-dependent physical response, we can conclude that a good isolation for different mode is realized theoretically. To prove our deduction, a novel method proposed recently^38^ is applied here to calculate the purity of OAM modes. The criterion to judge the purity of different OAM modes or the degree of distortion is based on the phase gradient method, as shown as Equation (4), where $l$ is the OAM mode, $N$ represents the sampling number, ${\varphi }_{n}$ is the phase of electrical field of the $n-th$ sampling point and ${\phi }_{n}$ is the azimuth angle of the $n-th$ sampling point.

4 $${\sigma }^{2}=\frac{{(\frac{\varphi 1-\varphi N}{\phi 1-\phi N}-l)}^{2}+\sum _{2}^{N}{(\frac{\varphi n-\varphi n-1}{\phi n-\phi n-1}-l)}^{2}}{N}$$

In our analysis, the sampling interval is 1 degree and *N* is 361. The sampling interface is 0.6 m away from the metasurface and its radius is 0.5 m. For different generated OAM modes, variances for the expected and unexpected modes are calculated and shown in Table 4. It can be found that a good purity of OAM mode has been achieved. For now, there is no widely accepted standard for determining OAM mode purity, and the results are sensitive to the number and location of sampling points, antenna design and the method for measuring.

**Table 4:** Variances for different OAM modes.

${\sigma }^{2}$	The mode of generated OAM wave	
	2	4
$l={\rm{2}}$	3.8188	17.9103
$l={\rm{4}}$	15.6344	2.3867

### Experimental validations

**Figure 13:**
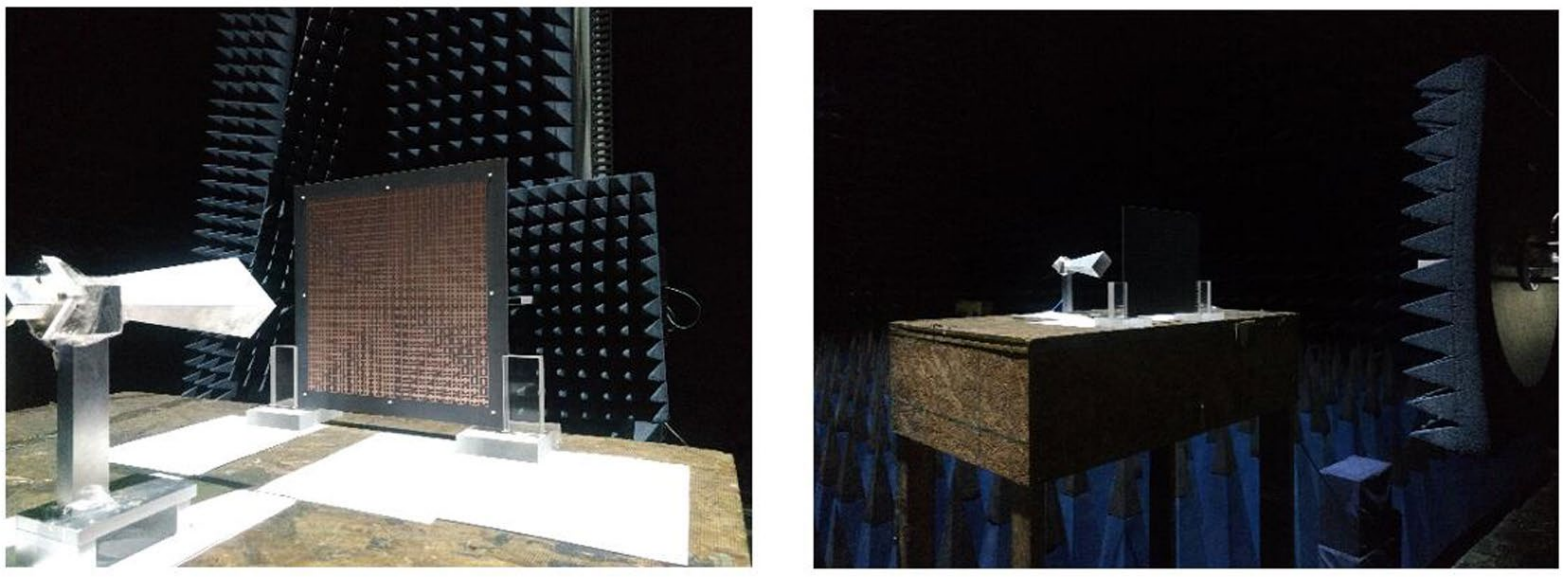
Fabricated OAM-generating system with dual polarization and dual mode.

**Figure 14:**
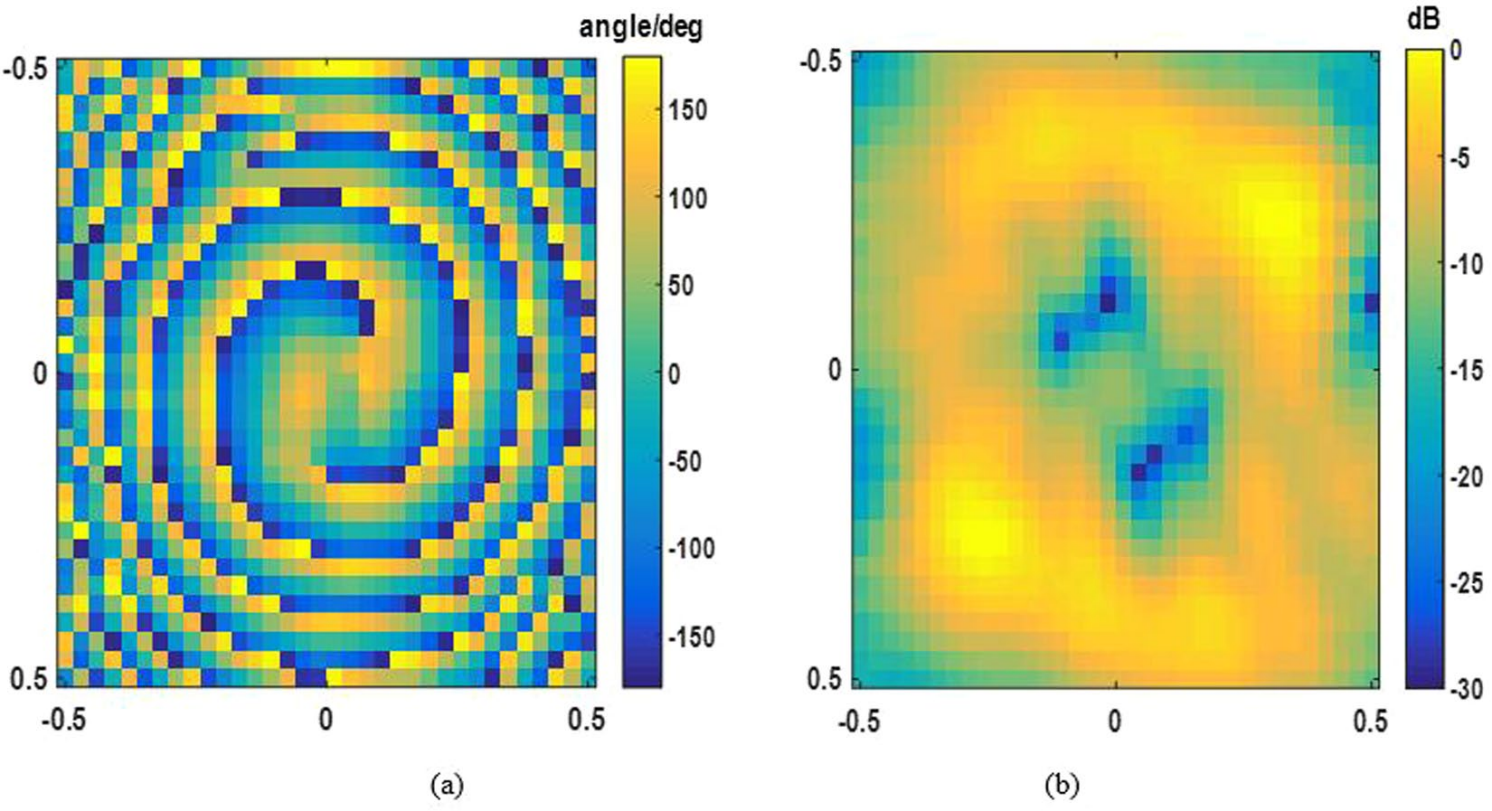
Measured x-polarization E-field distribution. (**a**) phase (**b**) magnitude.

**Figure 15:**
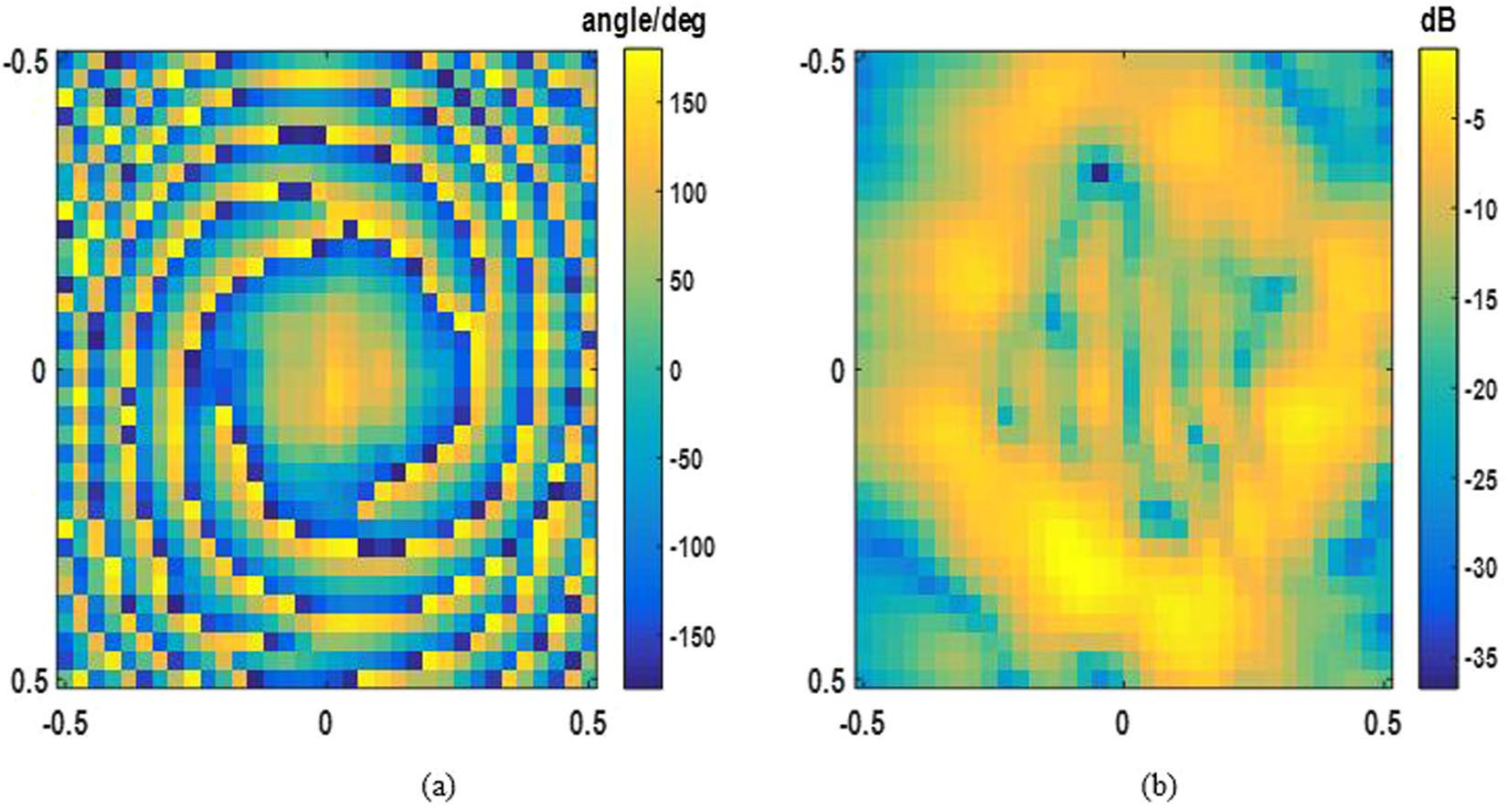
Measured y-polarization E-field distribution. (**a**) phase (**b**) magnitude.

For experimental demonstration, we fabricate and measure the OAM-generating system discussed above, which is illustrated in Fig. 13. Distributions of two orthogonal polarized electrical fields are measured by the near-field planar scanning technique. The operating frequency is 7.5 GHz and the near-field sampling plane with the dimension of 0.5 m*0.5 m is 0.6 m away from the metasurface. The number of sampling points is 33*33. The measured magnitudes and phases of x- and y-polarizations are shown in Figs 14 and 15, corresponding to OAM mode = 2 and mode = 4 respectively.

A good agreement between the simulated and measured results has been achieved. The spatial vortex distribution of phase can be identified clearly by Figs 14(a) and 15(a), and a typical magnitude null at the center of beam appears also, matching the numerical simulations. The experimental results are slightly different from those of simulations. The reason for the slight discrepancy between simulation and experimental results would be due to edge diffraction of the metasurface, dimension tolerance of the unit cells, and machining error for stacking.

**Figure 16:**
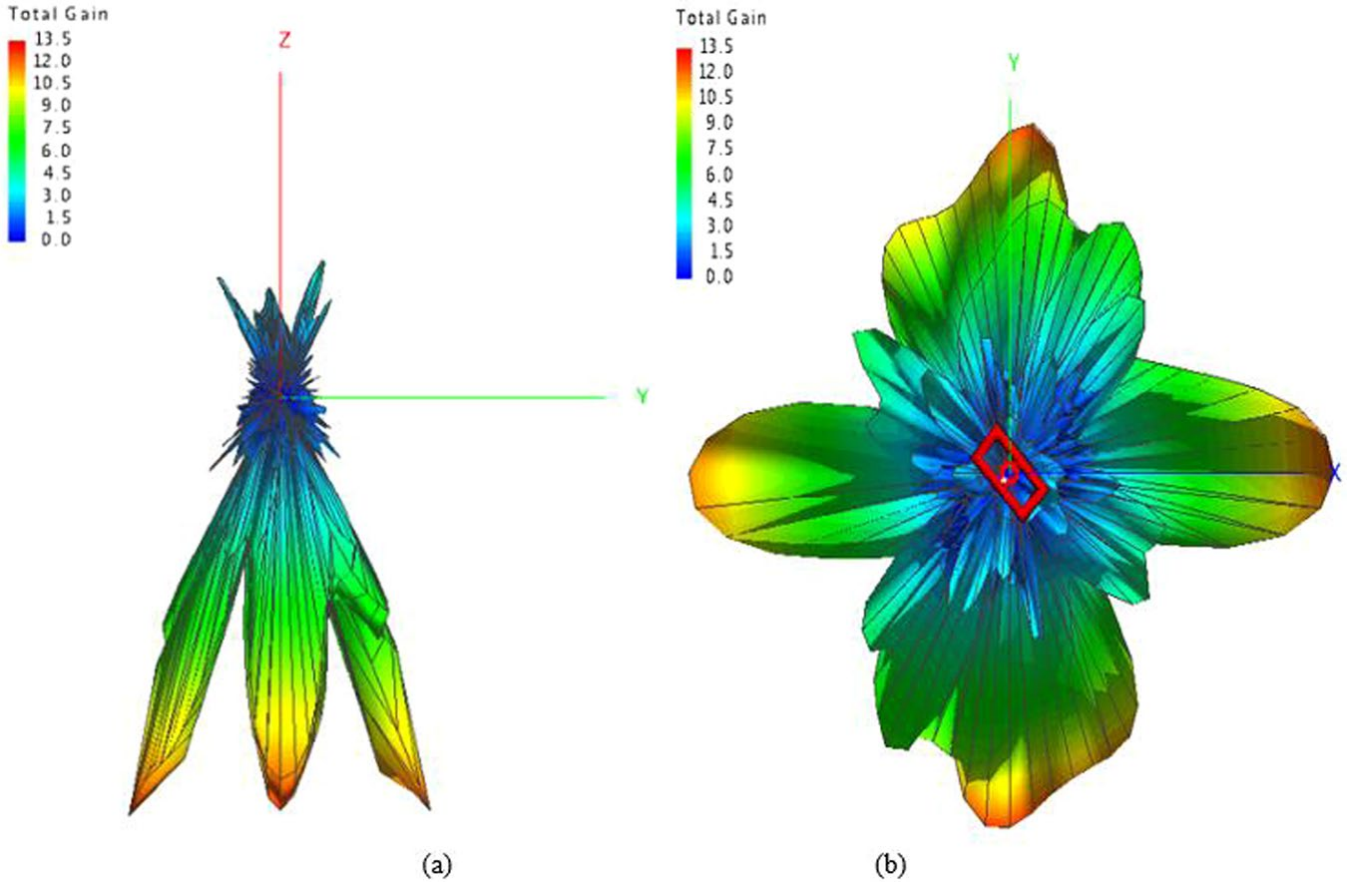
Simulated 3-D far-field radiation pattern (**a**) front view (**b**) top view.

**Figure 17:**
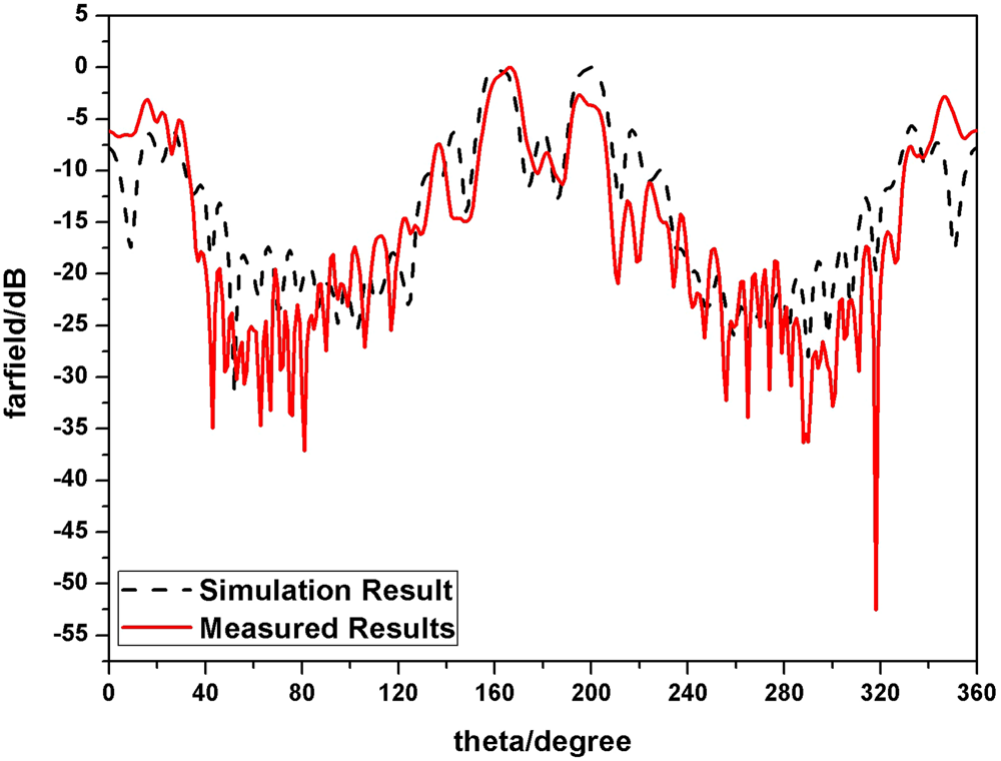
Simulated and measured 3-D far-field radiation patterns of y-o-z plane.

Furthermore, the simulated 3-D far-field radiation pattern is drawn in Figs 16, 17 compares the simulated and measured far-field radiation patterns of y-o-z plane. Both figures show the far-field radiation patterns comprising OAM mode of 2 and 4 simultaneously. A typical magnitude null at the center of the beam can be identified clearly and the simulated and measured results coincide well with each other.

## Discussion

In this paper, a practical dual-mode dual-polarization OAM generating system has been numerically and experimentally demonstrated by using a transmissive metasurface with optimized element structure. The element is well-designed and can introduce additional phase to the incident wave to generate EM waves carrying OAM. Dual-mode operating property is realized by using the polarization-dependent physical response. The transmissive metasurface provides a high-performance of transmission and a full 360° phase variation by employing four stacked layers. As expected, our numerical and experimental results show that the proposed transmissive metasurface is able to generate dual-mode dual-polarization OAM waves effectively and simultaneously. It’s noticeable that the presented design in this paper is for generating dual-polarization dual-mode EM waves carrying OAM, the same working mechanism and design strategy also can be adapted to generate dual-band dual-mode vortex waves, which enhances the system capacity further. The proposed OAM-generating system paves a way for practical application of OAM in modern wireless communications.

## Methods

Numerical simulations for element design are performed by the finite element method based commercial software CST. Numerical simulations for the whole metasurface-feed system are performed by the method of moments based on commercial software FEKO. The experimental structure is fabricated by using PCB technology with the substrate of F4B. The radiation pattern and near-field scanning are measured in a microwave anechoic chamber by NSI.
